# Reliability of generic quality-of-life instruments in assessing health-related quality of life among children and adolescents with idiopathic nephrotic syndrome: a systematic review

**DOI:** 10.1186/s12955-021-01786-w

**Published:** 2021-05-12

**Authors:** Ann E. Aronu, Samuel N. Uwaezuoke, Uzoamaka V. Muoneke

**Affiliations:** 1 Department of Pediatrics, University of Nigeria Teaching Hospital, Ituku-Ozalla, Enugu, 400001 Nigeria; 2 College of Medicine, The University of Nigeria, Ituku-Ozalla Enugu campus, Nigeria

**Keywords:** Adolescents, Children, Generic instruments, Health-related quality of life, Idiopathic nephrotic syndrome

## Abstract

**Introduction:**

Most of the studies reporting the negative impact of idiopathic nephrotic syndrome on health-related quality of life in children and adolescents were conducted with generic quality-of-life instruments rather than disease-specific instruments. The consistency of these studies' findings using these generic instruments is not well established.

**Aim:**

This systematic review aims to determine the reliability of current generic quality-of-life instruments in assessing health-related quality of life among children and adolescents with idiopathic nephrotic syndrome.

**Methods:**

We searched the PubMed, MEDLINE, EMBASE, and Google Scholar databases for articles published between 2000 and 2020, using appropriate descriptors. We included primary studies that met the eligibility criteria, independently screened their titles and abstracts, and removed all duplicates during the study-selection process. We resolved disagreements until a consensus was reached on study selection. We independently retrieved relevant data, including the generic quality-of-life instruments and the subjects’ and controls’ aggregate health-related quality of life scores, using a preconceived data-extraction form.

**Results:**

Ten original articles were selected for qualitative and quantitative analyses. Some of the studies reported the following significant findings. The mean health-related quality of life scores for children with prevalent and incident nephrotic syndrome were 68.6 (range, 52.6–84.6) and 73.7 (range, 55.9–91.5), respectively. Children with idiopathic nephrotic syndrome and their controls with other chronic diseases had median scores of 65 (interquartile range, 59–68.75) and 62.2 (interquartile range, 58.05–65.78). Patients on oral immunosuppressive drug and intravenous rituximab reportedly had median scores of 76.2 and 72.6 and mean scores of 71.4 (range, 55.4–87.4) and 61.6 (range, 42.1–81.1) respectively for quality-of-life assessment on the ‘school functioning domain.’

**Conclusions:**

The health-related quality of life scores in patients with idiopathic nephrotic syndrome are consistently low. Lower scores occur in prolonged disease duration and severe clinical phenotypes, whereas the scores are higher than the scores obtained in other chronic diseases. These consistent findings underscore the reliability of the current generic instruments in assessing health-related quality of life in patients with idiopathic nephrotic syndrome.

## Introduction

Idiopathic nephrotic syndrome (INS) is the most frequent manifestation of glomerular disease in children worldwide. Children with INS may face the challenges of frequent relapses, steroid-dependence, steroid-resistance, or resistance to other immunosuppressive drugs, and side-effects of these medications [1, 2]. Thus, INS usually runs a chronic course in children because of these challenges. Besides, some children who present with steroid-resistant nephrotic syndrome (SRNS) – especially cases due to focal segmental glomerulosclerosis (FSGS)—may end up with end-stage kidney disease (ESKD) and would ultimately require renal replacement therapy.

The chronicity of the disease potentially results in both physical and psychosocial strain on affected children. The considerable treatment burden and prognostic implications may also lead to psychological distress in their parents or caregivers [3]. Optimizing the care of children with chronic kidney disease (CKD) should entail managing psychosocial and developmental issues that will promote a seamless transition into adulthood [4]. Therefore, their quality of survival is regarded as necessary and has also become a fundamental focus of holistic health care [5]. Measures of patient-reported outcomes (PROs) can assess the attainment of patients’ feelings of well-being in traditional clinical interventions. Compared with clinicians' objective health measures, patients' self-evaluation of their health status appears to be more predictive of morbidity and mortality [6]. For instance, assessing the quality of life (QoL) scores in children with late CKD stages may improve their management and health-related quality of life (HRQoL) outcomes, as these scores can influence the clinician’s decisions on treatment options.

QoL scores can be obtained by patients' self-reports or by parents' proxy-reports if they are too young or too ill to volunteer information on domains related to physical and occupational function, psychological state, and social interaction, and somatic sensation. Several generic and few disease-specific QoL instruments are currently available. Some generic tools include Pediatric Inventory of Quality of Life (PedsQL) 4.0 Generic Core Scales [7], SF-36 [8], the Sickness Impact Profile (SIP) [9], Generic Children's QoL Measure (GCQ) [10], and the EuroQoL [11]. For instance, the Pediatric Inventory of Quality of Life (PedsQL) 4.0 Generic Core Scales have been used to assess the physical, emotional, social, school, and overall functioning of healthy children and adolescents and their counterparts with kidney-related diseases [7]. Given their non-specific nature, less objective findings may be obtained in affected patients. In contrast, kidney disease-specific instruments (currently few and non-validated) would likely generate more reliable data on patients’ feelings of well-being in the explored domains.

Nevertheless, studies conducted in children with ESKD consistently reported low HRQoL scores using the generic QoL instruments [12–14]. Other studies from developed and developing countries that utilized these tools reported similar findings in children with INS [15–17]. The scores were influenced by disease phenotypes and these instruments’ QoL domains. Given the dearth of kidney disease-specific QoL instruments, this systematic review aims to determine the reliability of the available generic instruments in assessing HRQoL among children and adolescents with INS. It was conducted and reported according to the Preferred Reporting Items for Systematic Reviews and Meta-Analyses (PRISMA) guidelines.

## Methods

### Search strategy

SNU, AEA, and UVM searched the PubMed, MEDLINE, EMBASE, and Google Scholar databases for articles published between 2000 and 2020. (Date of the last search: 19^th^ September 2020). The following descriptors were used, alone and in combination, for the search: ‘health-related quality of life,’ ‘patient-reported outcomes,’ ‘idiopathic nephrotic syndrome,’ ‘children,’ ‘adolescents,’ ‘quality-of-life outcome,’ and ‘generic instruments.’

### Eligibility and exclusion criteria

We included primary studies that met the following criteria: (i). observational studies of children without bias for race, socioeconomic, and educational background (ii). Full-text studies published in or translated into the English language (iii). Studies that utilized generic instruments and reported HRQoL in children with INS or HRQoL scores in children with ‘prevalent’ INS and their comparators with ‘incident’ disease or other chronic diseases. We excluded abstracts, letters to the Editor, reviews, commentaries, editorials, and studies without either primary data or described study methods.

### Study selection

SNU and AEA independently screened the titles and abstracts of retrieved published articles. Both authors obtained and further assessed potentially eligible full-text articles (both free and subscription-based) for final inclusion to the list of articles to be systematically reviewed. Both authors removed all duplicates during the study-selection process and resolved disagreements until a consensus was reached on selecting an eligible study.

### Quality assessment

The methodological quality of included studies was assessed using the Newcastle–Ottawa Scale (NOS) to assess non-randomized studies [18]. The NOS assesses case–control and cross-sectional studies using criteria grouped into ‘selection’ (maximum of 5 stars), ‘comparability’ (maximum of 2 stars), and ‘exposure/outcome’ (maximum of 3 stars). The star-rating was categorized as low if < 7 stars or high if ≥ 7 stars. SNU and AEA independently assessed the quality of included studies and resolved inter-rater discrepancies by consensus.

### Data extraction and data items

SNU and UVM independently retrieved relevant data from the selected studies using a preconceived data-extraction form. The form was designed to obtain information about the first author's name, year of publication, study setting and country, study design, study population, sample size, and demographics of study subjects such as age and sex distribution. Other extracted data were the generic HRQoL instruments used in each study and the study subjects' HRQoL scores and controls. The inter-rater reliability for selected qualitative items was measured using Cohen’s kappa coefficient (κ) [19].

### Synthesis of data

To establish the HRQoL-generic instruments' criterion validity, we compared the aggregate data on HRQoL scores (numerical indicators of HRQoL domains) in subjects and controls in some of the studies. This quantitative data synthesis was meant to objectively evaluate the impact of INS or its clinical phenotypes on the reduction of HRQoL scores compared to the scores in their healthy counterparts or those with ‘incident’ INS or those with other chronic diseases.

## Results

### Study selection

**Fig. 1:**
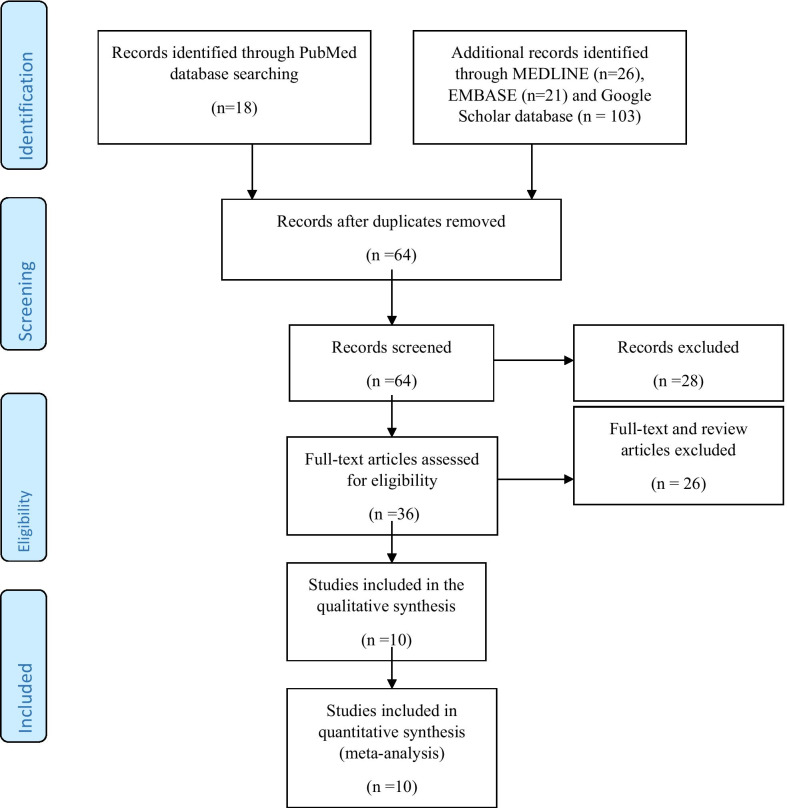
Algorithm for the inclusion of studies reporting health-related quality of life scores in children and adolescents with idiopathic nephrotic syndrome

We identified 18 relevant records in the PubMed database, 26 records in MEDLINE, 21 records in EMBASE, and 103 relevant records in the Google Scholar database, giving 168 records. After removing duplicates, the number of records was scaled down to 64. The remaining articles were then screened for relevance to the topic under review. Thirty-six records were left after this initial screening. Twenty-six records were further excluded leaving behind ten full-text original articles assessed for eligibility based on the inclusion criteria. These ten original articles were selected for qualitative and quantitative analyses in the present systematic review (Fig. 1). For these selected studies, Cohen’s kappa coefficient (κ) for qualitative items was estimated as 0.47 for inter-rater reliability, which we characterized as ‘fair to good’ based on Fleiss’s guideline [20].

### Study characteristics

The ten selected studies consist of case–control studies (n = 5) [10, 17, 21–23], longitudinal cohort studies (n = 2) [24, 25], and cross-sectional studies (n = 3) [15, 16, 26]. The countries of study were distributed among four continents as follows: North America [24, 25], Europe [16, 21], Asia [15, 17, 22, 23], and Africa [10, 26]. All the studies were hospital-based. A total of 1,011 subjects with INS were assessed in the ten studies. In the case–control studies, the total number of subjects and controls was 283 and 383, respectively. A total of 507 subjects participated in the two longitudinal cohort studies; 304 (60%) had ‘prevalent’ nephrotic syndrome (i.e., disease duration at baseline was ≥ 30 days) while 203 (40%) had ‘incident’ nephrotic syndrome (i.e., disease duration at baseline was < 30 days). Similarly, the total number of subjects for the cross-sectional studies was 221. In five studies that reported sex distribution [15, 17, 22, 24, 26], the total number of males and females was 204 and 122, respectively, with an approximate ratio of 1.7: 1 (Table 1). Study methodological quality, using the NOS, shows a star-rating of < 7 (low quality) for three studies [15, 16, 26] and ≥ 7 (high quality) for seven studies [17, 21–25]. (N/B: Rating indicates the range of quality [based on the adopted criteria from NOS] and does not translate to unreliable study findings for studies rated as low quality. Hence, all ten studies were reviewed on the same pedestal).

**Table 1:** Characteristics of the studies on health-related quality of life in children and adolescents with idiopathic nephrotic syndrome

Study (first author’s name and year of publication)	Country of study	Study setting	Study population (sample size and age/sex distribution)	Study design
Rüth et al. [21]	Switzerland	Zurich University hospital	45 subjects/controls Median ages at diagnosis: 9.8 years (3.4–19.8 years) for subjects and 5.9 years (0.1–16.3 years) for controls	Cross-sectional, case–control study
Selewski et al. [22]	United States	Multi-center setting (Midwest Pediatric Nephrology Consortium)	127 subjects (67 with prevalent NS and 60 with incident NS) Age range: 8–12 years (67 subjects) and 13–17 years (60 subjects) Male/Female (83 subjects/44 subjects)	Longitudinal cohort study
Rahman et al. [15]	Bangladesh	Dhaka Medical College Hospital	50 subjects Age range: 2–12 years (mean age: 7 ± 2.92 years) Male/Female (29 subjects/21 subjects)	Prospective cross-sectional study
Agrawal et al. [17]	India	Tertiary health facility in southern India	50 subjects and 50 controls Age range: 2–18 years Male/Female (30 subjects or controls/20 subjects or controls)	Cross-sectional, case–control study
Khanjari et al. 23]	Iran	Ali Asghar & Pediatric Medical Centers Mofid hospital, Tehran	38 subjects and 38 controls Age range: 8–12 years (mean age: 9.63 ± 1.49 years for subjects, 9.42 ± 1.51 years for controls) Male/Female (25/13 subjects and 26/12 controls)	A prospective, case–control study
Roussel et al. [16]	France	Pediatric Nephrology centers in France	110 subjects Age range:7–17 years (mean age: 11.6 years)	Cross-sectional observational study
Troost et al. [24]	United States Canada	Multi-center tertiary health-facility settings in the United States and Canada	56 subjects with incident INS* & 65 subjects with prevalent INS^‡^ (PROMIS-II) 87 subjects with incident INS & 172 prevalent INS (NEPTUNE) Age ranges: 8–17 years (children) > 18 years (adults)	Longitudinal cohort study (PROMIS-II and NEPTUNE)
Solarin et al. [25]	Nigeria	Tertiary health facility in Lagos	61 subjects Age range: 2–18 years (mean age: 5 ± 3.39 years) Male/Female (37 subjects/24 subjects)	A prospective, cross-sectional study
Jabbar et al. [26]	Iraq	Pediatric clinics at two hospitals in Baghdad	50 subjects/50 controls Age range:2–12 years	Prospective case–control study
Eid et al. [10]	Egypt	Mansoura University Children’s hospital	300 subjects (100 subjects with INS & 200 matched controls: healthy & chronic non-renal illness groups)	Prospective case–control study

PROMIS-II, Patient-reported outcomes measurement information systems II; NEPTUNE, Nephrotic syndrome study network consortium; INS, idiopathic nephrotic syndrome; *Disease duration at baseline < 30 days; ^‡^Disease duration at baseline ≥ 30 days

### Study findings

The individual studies reported the following significant findings, as shown in Tables 1 and 2. Among the case–control studies, Rüth et al. investigated 45 subjects and 45 controls whose median ages were 9.8 years and 5.9 years, respectively [21]. Child-and parent-rated QoL and psychosocial adjustment were evaluated in the study population, negatively impacting HRQoL and psychosocial adjustment in the subjects compared to the controls. Steroid dependency and cytotoxic therapy (which are surrogate indicators of INS chronicity) significantly negatively impacted HRQoL, while the family climate, such as maternal distress, negatively affected HRQoL and psychosocial adjustment. Agrawal et al. evaluated the HRQoL of 50 subjects and 50 comparators aged 2–18 years [17]. The comparators were age-and sex-matched children with other chronic illnesses (unspecified by the authors). The overall QoL scores were significantly higher in the INS subjects than in the comparators: especially in the physical, emotional, and social functioning domains. Both intervention groups, however, had similar scores on school performance.

**Table 2:** Health-related quality of life assessment in children and adolescents with idiopathic nephrotic syndrome: the generic instruments and the significant findings

Study (first author’s name and year of publication)	Study aims	HRQoL instruments	Evaluated parameters	Major findings
Rüth et al. [21]	To evaluate QoL and psychosocial adjustment by standardized tests	TNO-AZL Child Quality of Life Questionnaire Child Behavior checklist Teacher Report form	Child-and parent-rated QoL Child’s psychosocial adjustment	Child-rated QoL = 1/7 (14.3%) Parent-rated QoL = 4/7 (57.1%) Impairment of the child's psychosocial adjustment at home and school
Selewski et al. [22]	To evaluate the influence of disease duration on HRQoL and compare the differences in HRQoL in children with prevalent and incident INS To compare the findings of the PROMIS and PedsQL instruments	PROMIS II instrument Pediatric Quality of Life Inventory™ (PedsQL™) 4.0 Generic Scales	Child-rated QoL in both instruments’ domains	PROMIS scores significantly worse in prevalent than in incident INS for ‘pain interference’ and ‘peer relationships’ domains PedsQL scores significantly worse in prevalent than incident INS for ‘social functioning’ and ‘school functioning’ domains
Rahman et al. [15]	To evaluate the HRQoL in children with INS	Pediatric Quality of Life Inventory™ (PedsQL™) 4.0 Generic Scales Pediatric Quality of Life Questionnaire for the nephrotic syndrome (parental proxy-report)	Child-and parent-rated QoL	QoL significantly impaired, especially in physical and social summary scores
Agrawal et al. [17]	To compare the HRQoL in children with INS and children with other chronic illnesses (controls)	Pediatric Quality of Life Inventory™ (PedsQL™) 4.0 Generic Scales	Child-rated QoL	Aggregate QoL scores in children with INS better than in those with other chronic illnesses
Khanjari et al. [23]	To investigate the effect of blended training on HRQoL in children with INS	Pediatric Quality of Life Inventory™ (PedsQL™) 4.0 Generic Scales	Child-rated QoL	QoL scores increased in the intervention group compared to the control group after blended training
Roussel et al. [16]	To describe HRQoL in children with SDNS or SRNS on oral immunosuppressive treatment or intravenous RTX in stable remission	A 30-item standardized questionnaire with a global score of 0–100	Child-rated QoL^‡^	High global QoL score in 'difficult-to-treat' INS patients in stable remission on oral immunosuppressive or RTX treatment
Troost et al. [24]	To identify HRQoL profiles in children and adults with NS to improve the interpretability and clinical utility of PROMIS®	PROMIS II instrument	Child-and adult-rated QoL^†^	Complete proteinuria remission, reduction in symptoms, and shorter disease duration were significant predictors of better HRQoL profile membership
Solarin et al. [25]	To assess HRQoL in children with idiopathic nephrotic syndrome	Pediatric Quality of Life Inventory™ (PedsQL™) 4.0 Generic Scales	Child-and parent-rated QoL	Good overall QoL in children with INS but lower QoL in those with SRNS, CKD, and prolonged duration of illness
Jabbar et al. [26]	To assess HRQoL in children with idiopathic nephrotic syndrome about children with other chronic diseases	Pediatric Quality of Life Inventory™ (PedsQL™) 4.0 Generic Scales	Child-rated QoL	HRQOL scores in all domains were significantly higher in children with idiopathic nephrotic syndrome compared to those with chronic disease
Eid et al. [10]	To evaluate HRQoL in children with idiopathic nephrotic syndrome compared to healthy children and children with chronic non-renal diseases	Pediatric Quality of Life Inventory™ (PedsQL™) 4.0 Generic Scales Generic Children's QoL Measure (GCQ)	Child-rated QoL	Significantly higher mean PedsQL scores in the idiopathic nephrotic syndrome group compared to the chronic non-renal illness group but significantly lower compared to the healthy control group Significantly higher mean GCQ scores in the idiopathic nephrotic syndrome group compared to the chronic non-renal illness group and healthy control group

HRQoL, health-related quality of life; QoL, quality of life; TNO-AZL, The Netherlands Organization for Applied Scientific Research Academical Medical Center; PROMIS®, Patient-Reported Outcomes Measurement Information System®; SDNS, steroid-dependent nephrotic syndrome; SRNS, steroid-resistant nephrotic syndrome; RTX, rituximab; ^‡^ QoL on physical and emotional well-being, self-esteem, family, friends, school and disease; ^†^ To predict HRQoL profile membership

In another study by Khanjari et al. [22], 38 subjects (who had routine interventions and additional training for nephrotic syndrome) and 38 controls (who had only the routine interventions) were assessed for the effect of the blended training on child-rated HRQoL in patients with INS. HRQoL scores increased in the intervention (subjects) group compared to the control group after the blended training. Jabbar et al. assessed the child-rated HRQoL in 50 subjects with INS and 50 controls with other chronic diseases [23]. HRQoL scores in all domains were significantly higher in the subjects than in the controls. Eid et al. evaluated HRQoL in 100 children with INS compared to 100 healthy children and 100 children with chronic non-renal diseases [10]. The study was a child-rated HRQoL assessment, which showed two outcomes. The first outcome showed significantly higher mean PedsQL scores in the ‘nephrotic syndrome group’ than in the ‘chronic non-renal illness group’ but significantly lower scores than in the ‘healthy control group.’ The second outcome showed significantly higher mean Generic Children's QoL (GCQ) scores in the 'nephrotic syndrome group’ than in the ‘chronic non-renal illness group’ and the ‘healthy control group.’

In the two longitudinal cohort studies, patients with ‘prevalent’ and ‘incident’ nephrotic syndrome were assessed from different perspectives. Selewski et al. evaluated the influence of disease duration on HRQoL. They compared the differences in HRQoL in patient cohorts with ‘prevalent’ nephrotic syndrome (67 children) and ‘incident’ nephrotic syndrome (60 children), as well as the outcomes from using two QoL generic instruments [24]. The main findings were the significantly worse patient-reported outcomes measurement-information systems (PROMIS) scores in ‘prevalent’ than in ‘incident’ nephrotic syndrome for ‘pain interference’ and ‘peer relationships’ domains, and the significantly worse PedsQL scores in ‘prevalent’ than ‘incident’ nephrotic syndrome for ‘social functioning’ and ‘school functioning’ domains. On the other hand, Troost et al. evaluated HRQoL profiles in children and adult cohorts with nephrotic syndrome to improve the interpretability and clinical utility of PROMIS [25]. In the PROMIS II cohort, 56 patients with ‘incident’ INS and 65 patients with ‘prevalent’ INS were involved. The nephrotic syndrome study network consortium (NEPTUNE) cohort comprised 87 patients with ‘incident’ disease and 172 patients with the ‘prevalent’ disease. The authors found complete disease remission, reduced symptoms, and shorter disease duration as significant predictors of better HRQoL-profile membership.

The following findings were observed in the individual cross-sectional studies. Firstly, the study by Rahman et al. used the child-and parent-rated QoL assessment to evaluate the HRQoL of 50 nephrotic children [15]. The primary outcomes were low HRQoL scores, especially in the ‘physical’ and ‘social’ domains. Prolonged disease duration and frequent relapses contributed significantly to low HRQoL scores. Secondly, Roussel et al. described the HRQoL in 110 children with difficult-to-treat nephrotic syndrome on stable remission, on either oral immunosuppressive drugs or intravenous rituximab (RTX) [16]. Using a child-rated QoL evaluation, they found a high global HRQoL score on these parameters: physical and emotional well-being, self-esteem, family, friends, school, and disease. Finally, Solarin et al. assessed the HRQoL in 61 children with INS using child and parent ratings [26]. The study findings comprised a high overall HRQoL score in INS but a lower score in SRNS, CKD, and prolonged disease duration.

### Generic quality-of-life instruments used for QoL assessment

In the ten reviewed studies, the Pediatric Quality of Life Inventory (PedsQL™ 4.0 Generic Core Scales) was the most frequently employed generic QoL instrument for assessing QoL in children with INS; seven studies used it either alone [17, 22, 23, 26], or in combination with other instruments such as Pediatric Quality of Life Questionnaire for nephrotic syndrome [15], PROMIS instrument [24], and Generic Children's QoL Measure (GCQ) [10]. The remaining three studies used the following instruments alone in evaluating the HRQoL of the patients: Netherlands Organization for Applied Scientific Research-Academical Medical Center (TNO-AZL) Child Quality of Life Questionnaire, Child Behavior checklist, and Teacher Report form [21]; 30-item standardized questionnaire with a global score of 0–100 [17]; and PROMIS instrument [25]. The PedsQL™ 4.0 Generic Core Scales was used to evaluate child-rated HRQoL in six studies [10, 16, 17, 22–24], and both child- and parent-rated HRQoL in one study [26]. The PedsQL™ 4.0 Generic Core Scales specifically evaluates the HRQoL in five domains: physical functioning (eight items), psychosocial functioning, including emotional functioning (five items), social functioning (five items), and school functioning (five items). The PedsQL scores range from 0 to 100 points. The PROMIS pediatric measures include depression, anxiety, social-peer relationships, pain interference, fatigue, and mobility domains. Higher scores indicate higher levels of the domain consistent with the measure's name, signifying worse symptoms of depression, anxiety, fatigue, and pain interference and better functioning for mobility and peer relationships.

### Aggregate numerical indicators of QoL domains in some of the studies

**Fig. 2:**
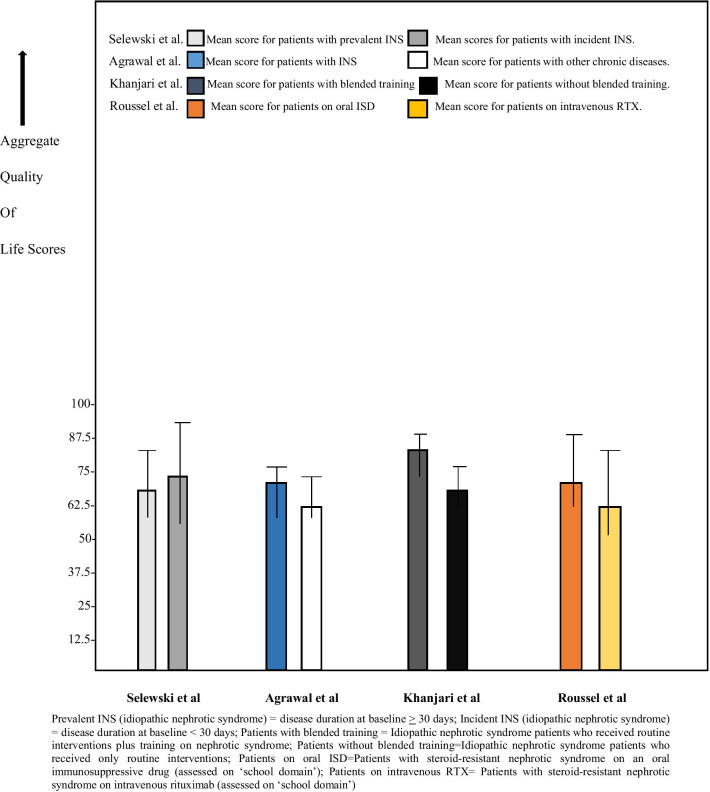
Aggregate health-related quality of life (HRQoL) scores in cohorts with idiopathic nephrotic syndrome reported in four studies

We could access the data indicating the aggregate numerical indicators of QoL domains in four studies [16, 17, 22, 24]. As shown in Table 3 and Fig. 2, the median or mean HRQoL scores in QoL domains reported in the four studies were computed and compared in our quantitative analysis. In the longitudinal cohort study by Selewski et al. [24], the mean HRQoL scores of children with ‘prevalent’ and ‘incident’ nephrotic syndrome were 68.6 (range: 52.6–84.6), and 73.7 (range: 55.9–91.5), respectively; showing significantly lower scores for prolonged (‘prevalent’) INS. The cross-sectional case–control study by Agrawal et al. reported median HRQoL scores of children with INS and their controls with other chronic diseases as 65 (interquartile range, 59–68.75) and 62.2 (interquartile range, 58.05–65.78) respectively [17]. Although both groups had low scores, the median score for children with INS was higher. Furthermore, the mean HRQoL scores of 65.5 (range: 52.9–78.1) and 78.1(range: 69.6–86.6) were documented by Khanjari et al. before and after a blended training for children with INS [22]. The study's control arm, who were children with the disease that received routine interventions, recorded mean scores of 64.5 (range: 56–73) and 65.1(range: 57.4–72.8) before and after the interventions. The blended training (comprising training on nephrotic syndrome and the routine interventions) improved the HRQoL scores, although they were low in both study arms. Finally, the cross-sectional observational study of children with steroid-dependent nephrotic syndrome (SDNS) or SRNS by Roussel et al. reported mean global HRQoL scores of 76.2 and 72.6 in patients on oral immunosuppressive drug and intravenous rituximab, respectively [16]. The mean scores of 71.4 (range: 55.4–87.4) and 61.6 (range: 42.1–81.1) were reported respectively in patients on oral immunosuppressive drug and intravenous rituximab for QoL assessment on the ‘school functioning’ domain.

**Table 3:** Aggregate health-related quality of life (HRQoL) scores in some individual studies

Study authors	Study design	HRQoL score (median or mean/SD score)	HRQoL score (median or mean/SD score)
Selewski et al. [22]	Longitudinal cohort study	68.6 ± 16.0*	73.7 ± 17.8**
Agrawal et al. [17]	Cross-sectional case–control study	65^†^, interquartile range = 59–68.75	62.2^††^, interquartile range = 58.05–65.78
Khanjari et al. [23]	Prospective case–control study	65.5 ± 12.6^‡^	64.6 ± 8.5^‡‡^
		78.1 ± 8.5^‡^	65.1 ± 7.7^‡‡^
Roussel et al. [16]	Cross-sectional observational study	76.2 ^¶^	72.6 ^¶¶^
		71.4 ± 16 ^§^	61.6 ± 19.5 ^§§^

*Mean score for children with prevalent nephrotic syndrome (disease duration at baseline ≥ 30 days) **Mean score for children with incident nephrotic syndrome (disease duration at baseline < 30 days) ^†^Median score for children with nephrotic syndrome ^††^Mean score for children with other chronic diseases ^‡^Mean scores for children with nephrotic syndrome before and after blended training ^‡‡^Mean scores of children with nephrotic syndrome before and after routine interventions ^¶^Mean global score for nephrotic patients on oral immunosuppressive drug ^¶¶^Mean global score for nephrotic patients on intravenous rituximab ^§^Mean score on ‘school functioning domain’ for nephrotic patients on oral immunosuppressive drug ^§§^Mean score on ‘school functioning domain’ for nephrotic patients on intravenous rituximab, SD, standard deviation

## Discussion

INS usually runs a chronic course in pediatric patients and may eventually end up in ESKD. Whereas children and adolescents with ESKD have been evaluated with generic QoL instruments and consistently found to have low HRQoL scores, those with INS should similarly have low scores, which could be influenced by disease- and patient-related factors. Evaluating the HRQoL in these patients using the current generic instruments will enable the use of PROs to make clinical decisions on management options. Because there is presently a dearth of kidney disease-specific QoL instruments, it is also important to establish the reliability of the available generic instruments in assessing HRQoL among these patients with INS.

In this systematic review, we qualitatively and quantitatively analyzed the aggregate numerical indicators of HRQoL domains in four studies [16, 17, 22, 24]; and found that the average HRQoL scores of children and adolescents with INS were significantly low. The low scores reported in these studies were variable. Disparity of scores was determined by disease duration [24–26], disease severity [16, 26], comparison with other non-renal chronic diseases [10, 18, 24], patient-rating of particular HRQoL domains [15, 16, 21, 24], and improved knowledge about the disease [22]. For instance, the scores tended to be lower with prolonged disease duration and severe clinical phenotypes but higher than those associated with other chronic diseases. The low mean child-rated HRQoL scores (e.g., 68.6 and 73.7) noted in these patients are in tandem with the mean total scores of 73.98 and 69.77 documented in patients with ESKD and patients on dialysis, respectively [14]. The lower mean scores of 61.6, recorded in the ‘school functioning’ domain in the study among nephrotic pediatric patients on intravenous RTX [16], are also consistent with the lower mean scores of 66.91 and 62.34 reported respectively for ESKD and dialysis patients [13]. Based on these findings, we suggest that the PROs of disease in INS and ESKD patients are similar and would frequently align with their clinical judgment.

We found dissimilarities in the low HRQOL scores and the HRQoL domains of some generic instruments based on child’s and parent’s reports. For instance, in the TNO-AZL Child QoL questionnaire used by Ruth et al. [21], child-rating of low HRQoL was noted in only one subscale of the instrument. Parent-rating of a similar change in HRQoL was, however, seen in four subscales. In the study by Rahman et al. [15], evaluation with child-rated PedsQL™ 4.0 Generic Core Scales showed much lower HRQoL scores in the ‘physical functioning’ and ‘social functioning’ domains. Parent-rated Pediatric QoL Questionnaire for nephrotic syndrome used in the same study indicated a significant association of low HRQoL with frequent disease relapses and prolonged disease duration [15]. Again, Solarin et al. used the child-and parent-reported assessment with PedsQL™ 4.0 Generic Core Scales and found that mean comparison of HRQoL scores of parents and children in the ‘physical functioning’ and ‘social functioning’ domains were significantly different [26]. Whereas parent-rated HRQoL scores were significantly low in the ‘physical functioning,' ‘emotional functioning,' and ‘social functioning’ and ‘overall’ domains, the child-rated scores were low in the ‘physical functioning’ domain [26].

In contrast, the same study's child-rated HRQoL scores were significantly low in the ‘emotional functioning’ domain in CKD or decline in estimated glomerular filtration rate (eGFR). In children with chronic diseases such as INS, the clinical evaluation's focus is usually the impact of disease activity. The change in HRQOL scores may reflect the current state of disease activity and therapy and the cumulative psychosocial impact of the disease course, duration, and cumulative drug exposure or high drug dosage [24, 27]. It may be trite to mention that the PedsQL™ 4.0 Generic Core Scales evaluate physical, emotional, social, and school functioning from the child’s perspective [7, 28–30]. A proxy-report on these domains from a parental perspective may thus be slightly different. Since the child primarily bears the physical and emotional brunt of the illness, any psychometrics will reveal the child's feelings in representative domains early enough. On the other hand, disease duration and treatment burden appear to influence parents' predominant domains. However, given the convergent validity of generic instruments like PROMIS and PedsQL 4.0 Generic Core Scales [24], and the high test–retest reliability and Cronbach’s α demonstrated in the PROMIS instrument domains [31]; we infer that PROs generated from these instruments remain dependable guides for clinical decisions despite the disparities in HRQoL scores from the child’s and parent’s reports.

This systematic review has its limitations. Firstly, the few studies we reviewed precluded a robust meta-analysis, although quantitative and qualitative analyses were done. Consequently, we could not derive a summary measure to establish the statistical significance of the aggregate data. We could only analyze the aggregate numerical indicators of HRQoL scores in only four eligible studies. Secondly, our inter-rater reliability with Cohen's kappa coefficient (κ) value of 0.47 is adjudged sub-optimal. However, we believe it did not affect the accuracy of our analyzed data as the reviewed studies showed clear evidence of similar QoL outcomes: indicating the consistency of generic QoL instruments in evaluating HRQoL in pediatric patients with INS. Thirdly, the limited publication dates (2000–2020) for the eligible published studies could have excluded relevant primary data for the systematic review. Nevertheless, our choice of the time frame for study selection was predicated on reviewing more recent studies on the topic. Finally, the systematic review did not consider interventions for QoL of the study populations, although these interventions can affect QoL even when measured with a generic instrument.

Although disease-specific QoL instruments were once employed to assess medical treatments and make treatment decisions [32, 33], the use of generic instruments such as PedsQL [7], SF-36 [8], the Sickness Impact Profile (SIP) [9], and the EuroQoL [11] appears to be the current paradigm. The PROMIS instrument is an improvement in evaluating children with kidney diseases. Worse still, no ESKD-specific instrument for children has been developed, although a PedsQL 3.0 ESKD module recently advanced by some authors holds promise in this direction but requires validation [34]. It would be interesting to conduct future HRQoL studies in children with INS using such kidney disease-specific instruments. Thus, we recommend repeat systematic reviews based on these future studies.

## Conclusions

The current validated generic QoL instruments indicate that INS children and adolescents have significantly low HRQoL scores irrespective of the study's geographical setting. More importantly, our analyzed aggregate numerical indicators of HRQoL domains, which consistently showed low HRQoL scores, objectively support the utility of these patient-reported outcome measures in driving the clinician's decisions on treatment options. More importantly, we suggest that the consistency of the findings across the reviewed studies underscore the reliability of these generic QoL instruments in assessing HRQoL in pediatric patients with INS.

## Data Availability

Not applicable.

## Abbreviations

CKD: Chronic kidney disease
eGFR: Estimated Glomerular filtration rate
ESKD: End-stage kidney disease
FSGS: Focal segmental glomerulosclerosis
HRQoL: Health-related quality of life
INS: Idiopathic nephrotic syndrome
NOS: Newcastle–Ottawa Scale
PRO: Patient-reported outcomes
PROMIS: Patient-reported outcomes measurement information systems
QoL: Quality of life
SRNS: Steroid-resistant nephrotic syndrome
SSNS: Steroid-sensitive nephrotic syndrome
